# Amelioration of lipid peroxidation *in vivo* and *in vitro* by *Satureja khozestanica* essential oil in alloxan-induced diabetic rats

**DOI:** 10.1186/s40200-014-0119-9

**Published:** 2014-12-13

**Authors:** Hassan Ahmadvand, Majid Tavafi, Ali Khosrowbeygi, Gholamreza Shahsavari, Maryam Hormozi, Khadijeh Beyranvand, Shahrokh Bagheri, Foad Abdolahpour

**Affiliations:** Razi Herbal Researches Center, Lorestan University of Medical Sciences, Khoram Abad, Iran; Department of Biochemistry, Faculty of Medicine, Lorestan University of Medical Sciences, Khoram Abad, Iran; Department of Anatomy, Faculty, Lorestan University of Medical Sciences, Khoram Abad, Iran

**Keywords:** Diabetes, Lipid peroxidation, Rat, *Satureja khozestanica*, Essential oil

## Abstract

**Background:**

We examined possible protective effect of *Satureja khozestanica* essential oil (SKE) on in *vivo* and *in vitro* lipid peroxidation in alloxan-induced Type 1 diabetic rats.

**Methods:**

Thirty Sprage-dawley male rats were divided into three groups randomly; group one as control, group two diabetic untreatment, and group three treatments with SKE by 500 ppm in drinking water, respectively. Diabetes was induced in the second and third groups by alloxan injection subcutaneously. After 8 weeks, animals were anaesthetized, livers and kidneys were then removed immediately and used fresh or kept frozen until their lipid peroxidation analysis. Lipid peroxidation was determined by measurement of thiobarbituric acid reactive substances (TBARS). Blood samples were also collected before killing to measure the levels of fasting blood suger (FBS) and lipid peroxidation.

**Results:**

SKE significantly inhibited the levels of FBS, TBARS serum and kidney content in treated group compared with the diabetic untreated group. Also the levels of malonedialdehyde liver content unaltered in treated group. SKE significantly inhibited LDL oxidation *in vitro*.

**Conclusions:**

The findings showed that SKE exerts beneficial effects on the lipid peroxidation in alloxan-induced Type 1 diabetic rats.

## Background

Free radicals are generated continuously in the body due to both normal metabolism and disease [1]. Oxidative stress is the imbalance between oxidant and antioxidant systems in favor of the former. Antioxidant systems include antioxidant enzymes such as superoxide dismutase (SOD), catalase (CAT) and glutathione peroxidase (GPX), in addition to low molecular agents and dietary antioxidants. Clinical and experimental studies have shown that disturbing of oxidant–antioxidant balance system is involved in the pathogenesis of chronic diseases such as cancer [2], coronary heart disease, diabetes and many diabetic complications [2]. Hyperglycemia is confounded for the complications of diabetes because hyperglycemia directly causes glycation of proteins, lipids and nucleic acid, then injures cells and induces lipid dperoxidation [3]. Also antioxidant and antioxidative enzyme activities reduce due to glycation or increase of lipid peroxidation products [4]. A number of natural antioxidant such as vitamin E and phenolic compunds are known to have hypoglycaemic, hypolipidemic or both activities [5]. Chemical drugs have many side effects; therefore, screening for new antidiabetic sources from natural antioxidants is still attractive because they are safe and good alternrtive for treatment of diabetes mellitus. A growing body of research indicates that nutritional deficiencies such as antioxidants contribute to the development of diabetes. Recently, much attention has been focused on the central and key role of oxidative stress in the pathogenesis of different diabetic complications [6]. Several studies have shown that antioxidant treatment reduces diabetic complications [7]. Because of increasing demand of patients for the use of natural products and other herbal drugs with anti-diabetic activity, the general trend now is to use the natural products for medicinal application in their natural available form [8]. Polyphenols, well-known antioxidants, have also been showed to function as antidiabetic by reducing blood glucose levels [9]. Researchers are recently interested in investigation and research into extraction of natural antioxidants from medical herbs to replace synthetic antioxidants [10]. Therefore, the research into the determination of the natural antioxidant source is very important to promote public health [10].

*Satureja-khuzestanica,* an endemic plant of Iran, decreases glucose and malondealdehyde in serum diabetic patients [10,11]. The components of this extract were analyzed with gas chromatography/mass spectrometry (GC/MS) in Research Center of Lorestan University as reported in our previous paper [11]. The main component of this extract is carvacrol as a good antioxidant [11].

Since the protective effects of SKE on lipid peroxidation status in alloxan-induced type 1 diabetic rats have not previously been reported; the objectives of the present study were to investigate amelioration of altered *in vitro* and *in vivo* lipid peroxidation status by *Satureja khozestanica* essential oil in alloxan-induced type 1 diabetic rats.

## Materials and methods

### Isolation of the essential oil from Satureja *khozestanica*

*Satureja khozestanica* essential oil was prepared from cultivated *satureja khozestanica* in Khoram Abad (Lorestan province, western Iran). The aerial parts of the plants were collected during flowering stage and were air-dried at ambient temperature in the shade. The aerial parts were hydro-distilled using a Clevenger apparatus for 4 hours, giving yellow oil in 0.9% yield. The oil was dried over anhydrous sodium sulfate and stored at 4°C. The plant was previously identified by the Department of Botany of the Research Institute of Forests and Rangelands (TARI) in Tehran, Iran. A voucher specimen (No. 58416) has been deposited at the TARI Herbarium [10]. The components of *Satureja khozestanica* essential oil were analyzed with gas chromatography/mass spectrometry (GC/MS) in Research Center of Lorestan University (Table 1).

**Table 1:** **The components of satureja khozestanica essential oil that analyzed by GC/MS**

**No**	**Compound name**	**Area (%)**	**No**	**Compound name**	**Area (%)**
1	3-Methyl butanol	0.14	19	β-Phellandrene	0.34
2	Eugenol	1.33	20	α -Thujene	1.26
3	1,8-Cineole	0.24	21	β -Caryophyllene	0.7
4	α -Pinene	0.99	22	γ-Terpinene	2.77
5	Geranyl acetone	0.5	23	Camphene	0.14
6	cis-Sabinene hydrate	0.68	24	α-Franesene	0.7
7	iso-Amylpropionate	0.23	25	Terpinolene	0.22
8	β-Bisabolene	3.77	26	β-Pinene	0.32
9	Linalool	3.32	27	α-Bisabolene	0.51
10	Myrcene	2.43	28	Nonanal	0.24
11	Caryophyllene oxide	1.53	29	trans-2-Carene-4-ol	0.73
12	4-Terpineol	4.1	30	β-Udesmol	0.32
13	iso-Butyl-2-methyl butyrate	0.19	31	α-Terpineol	0.42
14	Heptadecane	0.19	32	3-Carene	0.36
15	Thymyl methyl ether	1.21	33	α-Bisabolol	0.27
16	α-Terpinene	0.73	34	trans-Dihydrocarvone	0.26
17	Musk ambrette	0.08	35	para-Cymene	5.61
18	Carvacrol	63.17			

### Animals

Thirty male mature Sprague–Dawley rats (180–200 g) were obtained from Pasteur Institute of Tehran and were allowed to adapt themselves with the new location for one week. This study was approved by the Animal Ethics Committee of the Medical University of Lorestan with accordance to the national health and medical research council guidelines. The rats were divided to tree groups (10 per each). The studied groups were as follows: group 1 as control, group 2 as diabetic without treatment and 3rd group as diabetic treatment with SKE.

### Diabetes induction

Diabetes was induced after overnight fasting in the second and third groups by injection of alloxan monohydrate (120 mg/kg) subcutaneously [10]. Beta cell degradation by alloxan leads to release of more insulin. Because of acute hypoglycemia, the rats received 10% sucrose solution for 48 hr instead of drinking water. Five days after induction of diabetes, blood samples were gathered from the end part of tails. Blood glucose was measured by glucometer and the rats with blood glucose level of ≥300 mg/dl (16.7 mmol/l) were considered as diabetic [10,12]. During the first five days after diabetes induction, 1–3 rats per group died because of alloxan toxicity. The rats were kept at 12/12 dark–light period in 21 ± 3°C temperature. All animals were allowed free access to food and water *ad libitum* during the experiment. The third group was treated with SKE by 500 ppm in drinking water [10]. The treatment was begun at the first day of diabetes induction. After 8 weeks treatment, animals were anesthetized (Nesdonal 50 mg/kg, i.p.), blood samples were obtained from hearts and allowed to clot for 20 minutes in laboratory temperature and then centrifuged at 3000 rpm for 10 minutes for serum separation [10]. Also, then liver and kidneys were removed immediately and used fresh or kept frozen until the analysis.

### Levels of FBS

FBS was measured by biochemical analyzer using commercial kits (Olympus AU-600, Tokyo, Japan).

### Levels of TBARS

The serum levels of TBARS as a product of lipid peroxidation which reacts with thiobarbituric acid (TBA) analyzed. Also, then liver and kidney TBARS content were analyzed [13]. The absorbance was measured spectrophotometrically at 532 nm and the concentrations were expressed as nmol TBARS /mg-pr.

### *In vitro* oxidation of LDL

Blood samples were taken from healthy human and then serum was prepared. The LDL fraction was isolated from fresh serum by single vertical discontinuous density gradient ultracentrifugation [14]. Lipid peroxidation end products were determined as TBARS according to modified method of Buege and Aust [13]. After isolation of total LDL, the protein content of LDL was measured according to method of Bradford [15]. LDL was adjusted to 150 μg/ml of LDL protein with 10 mM PBS, pH7.4 and then aliquots of SKE were added to the solution. After initiating the oxidation process with CuSO_4_, the sample mixtures were incubated at 37°C for 5 h in a water bath and the reaction was terminated by adding EDTA (2 mM). TBARS formation was measured in a spectrophotometer at 532 nm. The results were recorded as TBARS equivalent content (nmol/mg LDL-protein) (1.56 * 10^5^ M^−1^. cm ^-1^) [13].

### Statistical analysis

All values are expressed as mean ± SD. The data were compared between groups by Mann–Whitney U test. Statistical analyses were performed using the SPSS 13 for windows software. A *P* value of < 0.05 was considered statistically significant.

## Results

### Effect of SKE on FBS of diabetic rats

**Figure 1:**
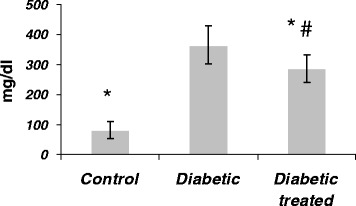
**The effect of SKE on serum FBS in alloxan induced diabetic rats.** *P < .05 as compared with control group. #*P* < .05 as compared with diabetic without treatment group.

The levels of FBS are shown in Figure 1. Diabetes significantly increased serum FBS in comparison with the control group. Treatment of diabetic animals with SKE significantly inhibited increase of serum FBS in comparison with the untreated diabetic animals

### Effect of SKE on serum TBARS of diabetic rats

**Figure 2:**
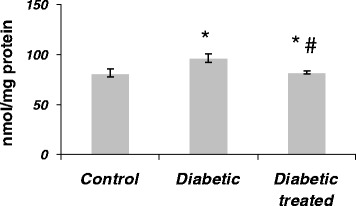
**The effect of SKE on serum TBARS in alloxan induced diabetic rats.** *P < .05 as compared with control group. #*P* < .05 as compared with diabetic without treatment group.

The levels of TBARS in serum are shown in Figure 2. The level of TBARS in the untreated diabetic rats was significantly (1.3-fold) higher than that of control animals. The level of TBARS in the serum of diabetic rats treated with SKE was very low, similar to the level found in the control animals. The treatment of diabetic animal with SKE could significantly (23%) inhibit the increase of TBARS in comparison with the untreated diabetic animals.

### Effect of SKE on kidney TBARS content of diabetic rats

**Figure 3:**
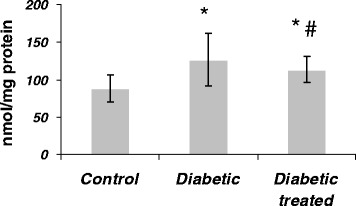
**The effect of SKE on kidney TBARS content in alloxan induced diabetic rats.** *P < .05 as compared with control group. #*P* < .05 as compared with diabetic without treatment group.

The level of renal TBARS in the untreated diabetic rats was significantly (1.4-fold) higher than that of control animals. The treatment of diabetic animal with SKE could significantly (33%) inhibit the increase of TBARS in comparison with the untreated diabetic animals (Figure 3).

### Effect of SKE on liver TBARS content of diabetic rats

**Figure 4:**
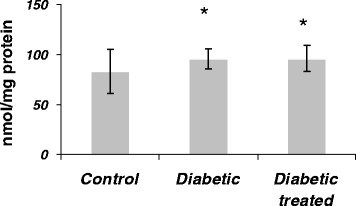
**The effect of SKE on Liver TBARS content in alloxan induced diabetic rats.** *P < .05 as compared with control group. #*P* < .05 as compared with diabetic without treatment group.

The level of liver TBARS in the untreated diabetic rats was significantly (1.16-fold) higher than that of control animals. The treatment of diabetic animal with SKE could (2%) inhibit the increase of TBARS in comparison with the untreated diabetic animals, but it was not significant (Figure 4).

### *In vitro* oxidation of LDL

**Figure 5:**
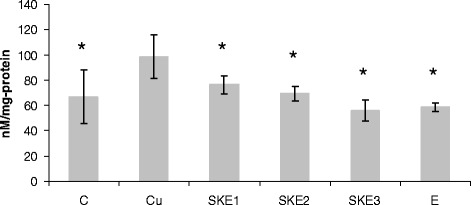
**The effects of SKE on the formation of TBARS. The effects of SKE on LDL oxidation in l0 mM PBS, pH 7.4 at 37°C for 5 h. (C) n-LDL, (Cu) n-LDL + copper, (SKE1) n-LDL + SKE (50 μg/m1), (SKE2) n-LDL + SKE (100 μg/m1), (SKE3) n- LDL + SKE (200 μg/m1) and (E1) n-LDL + Vitamin E(100 μM).** Each point represents the mean of five experiments.

The levels of TBARS after 5 h of incubation in all experiment groups are shown in Figure 5. Vitamin E significantly inhibited TBARS formation (*P* < 0.01). SKE exhibited a dose-dependent inhibition of TBARS formation. SKE (50, 100, 200 μg/m1) significantly was inhibited by the TBARS production in LDL (*P* < 0.01 and *P* < 0.001) respectively.

## Discussion

This study showed that SKE has protective effects on glomerular hypertrophy, glomerulosclerosis and loss of glomerular volume in alloxan-induced-diabetic rats. There is much evidence that oxidative stress play a key role in the most pathogenic pathway of diabetic injuries. Free radicals such as superoxide can induce cell and tissue injuries throughout lipid peroxidation and increase carcinogenesis, inflammation, early aging, cardiovascular diseases and tissue damage in diabetes [12]. Herbal extracts such as *Artemisia afra Jacq*, *Aframomum melegueta* and *Aloe vera* gel extracts [16] and antioxidants such as vitamin E, coenzyme Q10 and antioxidant enzymes such as SOD, GPX and CAT protect the cells against oxidative stress mediated cellular injuries by converting the toxic free radicals to non-toxic products[17]. Therefore use of antioxidant as complementary therapy is useful for diseases that related to oxidative stress.

### Effect of SKE on serum lipid peroxidation

Diabetes significantly increased serum, liver and kidney lipid peroxidation in comparison with the control group. Treatment of diabetic animals with SKE significantly inhibited decrease of serum and kidney lipid peroxidation in comparison with the untreated diabetic animals. There are reports that natural antioxidant such as vitamin E, vitamin C, lipoic acid, aminoguanidine, turine, N-acetylcysteine, allopurinollycopene and natural phenolic compounds have protective effects on lipid peroxidation in diabetics disease [18,19]. Many studies indicated that various herbal extracts have protective effects on lipid peroxidation in different disease. For example *aloe vera gel*, *Helicteres isora bark* extracts and grape seed *(Vitis vinifera)* extract extract ameliorate the oxidative stress in animal model [17].

Moreover; beneficial effects of SKE as antioxidant, antidiabetic, anti-inflammatory and anti-hyperlipidemia, toxicity and terato-genicity tests were also performed and confirmed plant’s safety [10]; moreover, this extract can be produced in large amount and low cost. Because of safety of SKE, a polyphenolic compound with antioxidant and anti-inflammatory properties, consumption of SKE is introduced to diabetic patients. Results of our study are in accordance with others researcher's study that showed SKE could reduce serum lipid peroxidation level. Therefore natural antioxidant with protective effects on lipid peroxidation could prevent or be helpful in reducing the complications that related to oxidative stress in diabetes patients.

#### *In vitro* antioxidant activity of SKE

The results clearly showed that various concentration of SKE have a dose-dependent antioxidant activity by inhibiting the formation of TBARS of LDL *in vitro.* The results showed that SKE especially at 200 μg/m1concentration similar to vitamin E as a positive control is a potent antioxidant and protect LDL in plasma against oxidation. Results of our study showed that SKE has beneficial effects, in decreasing lipid peroxidation *in vitro* and *in vivo*. Our results indicated that SKE is found to posses a good antioxidant activity. The difference in the amount of antioxidant of extracts may be attributed to the differences in the amount and kind of existing antioxidant compounds in them such as carotenoids, phenol and ascorbic acid [20].

The main constituents found in the SKE were Carvacrol (63.17%); para-Cymene (5.61%); 4-Terpineol (4.1%); β-Bisabolene (3.77%); Linalool (3.32%); γ-Terpinene (2.77); Myrcene (2.43%) (Table 1). The antioxidant activity has been shown by the SKE may be due to the presence of carvacrol, para-Cymene, 4-Terpineol, β-Bisabolene and Linalool [10,20]. Carvacrol is a good antioxidant-scavenger of peroxyl radicals and anti-inflammatory property [10,20]. Also researchers reported the role of oxidative stress as a central factor in onset and progression of diabetic complications such as vascular defects and nephropathy [5]. Inspite of numerous reports and our results that showed efficacy of antioxidative supplements administration in the prevention of diabetic complications. Since antioxidant therapy as one of the most important treatment strategies for diabetic patients for the prevention and slowing of diabetic complications progression such as hyperlipemia, hepatic damage. In diabetic nephropathy, structural injury develops over years before clinical and laboratory abnormalities such as albuminuria, hypertension, or declining glomerular filtration rate (GFR) appear. Thus, waiting for clinical or laboratory manifestation of renal disease before initiating treatment may hinder efforts that prevent progression to end stage of renal disease. Although the detailed molecular protective mechanisms of SKE can not be fully explained by our results, our results are satisfactory. SKE as a source of potent antioxidants. With multi beneficial properties can be introduced to diabetic patients without diabetic nephropathy for inhibition and progression of diabetic nephropathy.

## Conclusion

This study showed that SKE has protective effects on lipid peroxidation, glomerular hypertrophy, glomerulosclerosis and leukocyte infiltration in alloxan-induced-diabetic rats. Hence, attenuation of lipid peroxidation can decrease diabetic complication such as nephropathy in diabetic patients.
